# Autoimmune Hepatitis or Wilson’s Disease, a Clinical Dilemma

**DOI:** 10.5812/hepatmon.7872

**Published:** 2013-05-16

**Authors:** Melanie Deutsch, Theodoros Emmanuel, John Koskinas

**Affiliations:** 1 Academic Department of Internal Medicine, Hippokration General Hospital, Athens, Greece

**Keywords:** Autoimmune Chronic Hepatitis, Wilson Disease, Prednisolone, Azathioprine

*Dear Editor*,

The etiologic diagnosis of acute hepatitis and the correct therapeutic strategy may sometimes present several difficulties. We report the case of a young male patient with a severe acute hepatitis resembling features of both autoimmune hepatitis (AIH) and Wilson disease (WD). A 32-year old man presented with a seven days history of fatigue, low-grade fever and jaundice. The patient did not use tobacco, alcohol or illicit drugs. His parents and two siblings were in good health. Clinical examination revealed body temperature 37 ºC, blood pressure 130/80 mmHg, heart rate 72 beats/min and respiratory rate 20/min. The liver was slightly enlarged, painless, without tenderness on palpation, and with splenomegaly. He had no signs of chronic liver disease. Laboratory investigations revealed normal blood count, ESR 29 mm/1 h, AST 1219 IU/L, ALT 2327 IU/L, γGT 59 IU/L (normal values: 10-75 IU/L) and Alkaline Phosphatase 172 IU/L (normal values: 20-130 IU/L). Total Bilirubin was 29 mg/dL (direct 24 mg/dL) and Prothrombin time was 19 sec (INR: 2). Total proteins were 7.9 g/dL, Albumin 3 g/dL with Polyclonal Hypergammaglobulinemia. Serologic testing for viral hepatitis HAV, HBV, HCV, EBV, CMV, and HSV were negative. The antinuclear antibodies were positive (titer 1/1280). Serum Ceruloplasmin was 20 mg/dL (normal values 20-60 mg/dL), serum copper 390 μg/dL (normal values < 150 μg/dL), free copper 315µg/dL (normal values < 10 µg/dL) and urine copper 855 μg/24 h (normal values < 100 μg/24 h). Split lamp analysis revealed no Kayser-Fleischer ring. It caused the question which of acute AIH or WD would be the correct diagnosis. According to the international scoring system for AIH he had a score of 19. However, elevated serum copper, substantially high 24-hourr urine copper with Ceruloplasmin at the very lower normal limit suggested acute WD. The association of WD with autoimmune features and with AIH has rarely been documented (1, 2), and the difficulties in the differential diagnosis WD and AIH have been underlined especially in the pediatric population (3, 4). A liver biopsy showed interface hepatitis, portal invasion with mononuclear cell infiltrate and absence of fibrosis. Histochemical analysis with Rhodanine and Orcein was negative. The patient started treatment with Prednisolone 60 mg/day and Azathioprine 75 mg/day with clinical and biochemical improvement. At the remission phase, serum and urine copper levels were within normal limits. Molecular genetic analysis for mutations specific for WD (H1069Q, R1069Q, L936X, I1148T, and Q289X) was negative. Rhodanine histochemistry may be absent in early stages of WD (5) and the characteristic mutations are found in < 40% of patients. Kayser-Fleischer ring and history of neuro-psychiatric symptoms could be absent but 24 h urine copper remains abnormal in 80-85% of untreated patients with Wilson disease. On the other hand, alterations of copper metabolism may occur in the acute phase of severe icteric hepatitis (6), of any etiology, resulting in a misleading suspicion of WD (7). Urine copper levels, although occasionally increased in severe acute icteric hepatitis, does not exceed usually the value of 200 μg/24 h (855μg in our case) (8). After three years, the patient is in good health with normal liver biochemistry and continues on maintenance therapy with Prednisolone 2.5 mg/day and Azathioprine 75 mg/day. He didn’t develop other symptoms during follow up. This report shows the clinical challenge for the right diagnosis and therapy decision in a rare case of severe acute hepatitis resembling WD and/or AIH.
